# Adjustment of regional climate model output for modeling the climatic mass balance of all glaciers on Svalbard

**DOI:** 10.1002/2015JD024380

**Published:** 2016-05-21

**Authors:** Marco Möller, Friedrich Obleitner, Carleen H. Reijmer, Veijo A. Pohjola, Piotr Głowacki, Jack Kohler

**Affiliations:** ^1^ Department of GeographyRWTH Aachen UniversityAachenGermany; ^2^ Institute of Atmospheric and Cryospheric SciencesUniversity of InnsbruckInnsbruckAustria; ^3^ Institute for Marine and Atmospheric Research UtrechtUniversity of UtrechtUtrechtNetherlands; ^4^ Department of Earth SciencesUppsala UniversityUppsalaSweden; ^5^ Institute of GeophysicsPolish Academy of SciencesWarsawPoland; ^6^ Fram CentreNorwegian Polar InstituteTromsøNorway

**Keywords:** RCM output, glacier mass balance, Svalbard, modeling, Arctic

## Abstract

Large‐scale modeling of glacier mass balance relies often on the output from regional climate models (RCMs). However, the limited accuracy and spatial resolution of RCM output pose limitations on mass balance simulations at subregional or local scales. Moreover, RCM output is still rarely available over larger regions or for longer time periods. This study evaluates the extent to which it is possible to derive reliable region‐wide glacier mass balance estimates, using coarse resolution (10 km) RCM output for model forcing. Our data cover the entire Svalbard archipelago over one decade. To calculate mass balance, we use an index‐based model. Model parameters are not calibrated, but the RCM air temperature and precipitation fields are adjusted using in situ mass balance measurements as reference. We compare two different calibration methods: root mean square error minimization and regression optimization. The obtained air temperature shifts (+1.43°C versus +2.22°C) and precipitation scaling factors (1.23 versus 1.86) differ considerably between the two methods, which we attribute to inhomogeneities in the spatiotemporal distribution of the reference data. Our modeling suggests a mean annual climatic mass balance of −0.05 ± 0.40 m w.e. a^−1^ for Svalbard over 2000–2011 and a mean equilibrium line altitude of 452 ± 200 m  above sea level. We find that the limited spatial resolution of the RCM forcing with respect to real surface topography and the usage of spatially homogeneous RCM output adjustments and mass balance model parameters are responsible for much of the modeling uncertainty. Sensitivity of the results to model parameter uncertainty is comparably small and of minor importance.

## Key Points

- Optimal adjustment of RCM output varies depending on type of calibration due to inhomogeneous distribution of in situ reference data
- Mean annual climatic mass balance of all glaciers on Svalbard over 2000–2011 is −0.05 m w.e. a^−1^ with a mean ELA of 452 m asl
- Inaccurate RCM topography and inhomogeneous reference data distribution pose challenges to model calibration

## 1 Introduction

Regional climate model (RCM) outputs are often used as input data to calculate regional‐scale glacier mass balances [e.g.,*Fettweis*, 2007; *Machguth et al.*, 2009]. However, due to limitations in computational power and challenges in parameterizing model physics, the highest resolution RCMs generally only attain spatial resolutions of order 1–10 km when applied over extensive regions. They thus often fail to adequately reproduce local, terrain‐induced processes and conditions [e.g.,*Torma et al.*, 2015]. The simplest mass balance models are driven by air temperature and precipitation, but even those fields are often insufficiently reproduced in RCMs [e.g., *Franco et al.*, 2012]. Taking for granted the suitability of raw RCM output for glacier mass balance calculations is therefore not justifiable. Comprehensive in situ measurements are required to adjust relatively coarse resolution RCM output prior to mass balance modeling, as well as to validate calculation results [e.g., *Claremar et al.*, 2012; *Machguth et al.*, 2013]. In this study we concentrate on adjustments of air temperature and precipitation.

Two different kinds of in situ data can be used to obtain necessary RCM adjustments for mass balance modeling: records from automatic weather stations (AWS) and measurements of mass balances, either from stakes, snow pits, shallow firn cores, or snow radar. AWS output allows direct tuning of the adjustment parameters, although the lack of reliable precipitation measurements under cold conditions in remote areas without the possibility of using heated sensors poses challenges. However, AWS installations usually have limited spatial coverage compared to point mass balance measurements at stakes or snow pits. The latter data are less expensive and easier to obtain compared to AWS networks. On the other hand, their temporal resolution is generally limited to seasonal or annual values.

Accordingly, in this study we investigate whether it is possible to determine the optimal adjustment of coarse resolution RCM air temperature and precipitation output on the basis of point mass balance data only, neglecting more complicated subgrid parameterizations. In this respect, we focus on investigating the suitability of two different calibration approaches. We further investigate the extent to which coarse resolution mass balance estimates (10 km) adequately reproduce local conditions and whether they can thus be treated as a reliable representation of the regional‐scale glacier mass balance. This investigation is performed on all glaciers of the high Arctic archipelago Svalbard.

The glacierized area of Svalbard is 33,775 km^2^ [*Nuth et al.*, 2013], representing about 10% of all Arctic glaciers and ice caps apart from Alaska and Greenland but including Iceland [*Pfeffer et al.*, 2014]. There is high spatial heterogeneity across the archipelago with respect to both the degree of glacier coverage and the prevailing glacier types [*Hagen et al.*, 1993; *Nuth et al.*, 2013]. As a result, different minimal grid resolutions are required across the model domain to adequately reproduce the glacierized terrain. While higher‐resolution grids are necessary to represent smaller and steeper glaciers, lower spatial resolutions are sufficient for larger and flatter glacier areas.

The heterogeneous distribution of Svalbard's glaciers and ice caps is also reflected in their mass balance, which shows high spatial and temporal variability. *Nuth et al.* [2010] and *Moholdt et al.* [2010a] derived archipelago‐wide geodetic mass balances over regionally varying 15–40 year periods ending in 2005, while *Moholdt et al.* [2010b] did a comparable analysis for the more recent period of 2003–2008. These studies reveal considerable, nonsystematic, regional differences in ice mass evolution with time; an increasing gain of ice mass is evident at Austfonna, while the northwest Spitsbergen area as well as Barentsøya and Edgeøya continue to lose mass at comparable rates. In southern and northeastern Spitsbergen, the former ice mass loss has turned into an ice mass gain, while at Vestfonna, a development in the opposite direction has started. In western Svalbard, there is also evidence for increasing rates of ice mass loss [*Kohler et al.*, 2007].

Taken together, Svalbard has experienced a reduction of its ice mass loss over recent decades. *Moholdt et al.* [2010b] estimate a total mass change of ‐0.12 ± 0.04 m w.e. a^‐1^ (‐4.3 ± 1.4 Gt a^‐1^) for the period of 2003–2008, excluding changes of calving front positions. *Jacob et al.* [2012] estimate a change of ‐3 ± 2 Gt a^‐1^ from multitemporal GRACE (Gravity Recovery and Climate Experiment) satellite data sets for the period of 2003–2010, which includes calving front changes.

Modeling studies suggest a slightly negative mean surface mass balance for the glaciers of Svalbard, but the exact values vary. *Hagen et al.* [2003] calculated a mean rate of ‐0.013 ± 0.004 m w.e. a^‐1^ over the last three decades of the twentieth century, while a more recent RCM study [*Lang et al.*, 2015] yielded a mean rate of ‐0.047 m w.e. a^‐1^ for the period of 1979–2013.

**Figure 1:**
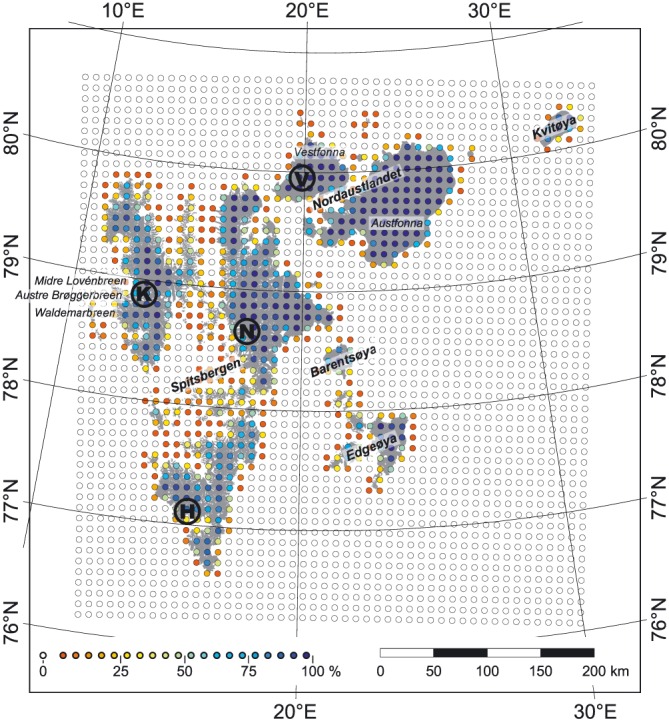
Overview map of the glacierized areas (dark grey) on the Svalbard archipelago. Locations of RCM grid points are indicated by colored circles. The color code indicates the degree of glacier coverage within a 10 × 10 km area centered at the respective RCM grid point. As each grid point represents an area of 100 km^2^, the percentages are equivalent to the area in km^2^. Areas of in situ mass balance measurements that are used as calibration sites are marked by black letters: Vestfonna (V), Kongsvegen (K), Nordenskiöldbreen (N), and Hansbreen (H). Names of individual glaciers and ice caps mentioned in the text are given in italics and those of the main islands of the archipelago in bold italics.

The outcome of our modeling study will complement this overall picture with an additional estimate for the period of 2000–2011. Here we use RCM output in the form of 10 km‐resolution air temperature and precipitation fields [*Finkelnburg*, 2013] to force our climatic mass balance (CMB) model. CMB is the sum of surface accumulation, surface ablation, and all refreezing [*Cogley et al.*, 2011]. As our study investigates the skill of straightforward adjustments of RCM air temperature and precipitation fields, we solely base our modeling architecture on these input variables and use a temperature index model for calculation of ablation. We use a set of several hundred mass balance stake readings from stakes located in four selected regions across the archipelago (Table 1 and Figure 1) to determine and evaluate the necessary RCM output adjustments. We then present time series of annual and seasonal CMB for all glaciers and ice caps on Svalbard, perform a thorough uncertainty assessment, and analyze the strengths and shortcomings of the approach used.

**Table 1:** Overview of Mass Balance Stakes and Readings Employed During the Cross Validation‐Based Adjustment of the RCM Output^a^

**Glacier**	**Subregion**	**Number of Stakes**	**Number of Readings**	**Elevation Interval**	**Time Pperiod**
Vestfonna	6	35	89	183–630	2007–2011
Kongsvegen	1	10	160	164–724	2000–2008
Nordenskiöldbreen	3	11	37	150–1150	2006–2011
Hansbreen	5	11	231	60–500	2000–2011

a Subregions are given according to Figure 7f. The elevation interval is the range of terrain elevations given in meter asl, where mass balance stakes are located on the particular glacier. The time period specifies the years from which stake readings are used.

## 2 Study Area

As stated, the degree and type of glacier coverage vary substantially across Svalbard [*Hagen et al.*, 1993]. Southern, northwestern, and eastern Spitsbergen, and parts of Barentsøya and Edgeøya, are covered by extensive ice fields, while Nordaustlandet and Kvitøya are dominated by large ice caps. In contrast, central Spitsbergen is distinctly less glacierized, with mostly cirque and valley glaciers. This pattern reflects in part the combined influences of precipitation and air temperature distribution across the archipelago as well as influences of local topography. Local glacier distribution can also be controlled by radiation effects [*Evans and Cox*, 2010] and by the influence of snowdrift [*Jaedicke and Gauer*, 2005; *Sauter et al.*, 2013]. Many outlet glaciers from the ice fields and ice caps terminate at sea, as calving tidewater glaciers [*Błaszczyk et al.*, 2009], and a large number of these glaciers are surge type [*Strozzi et al.*, 2008; *Sund et al.*, 2009; *Mansell et al.*, 2012].

The climate on Svalbard is controlled by the interaction of cold and warm air masses and ocean waters. The archipelago is situated at the intersection of warm and humid air coming from the Atlantic Ocean to the south and cold and dry Arctic air masses originating from the northeast [*Svendsen et al.*, 2002]. The West Spitsbergen Current represents the northernmost end of the North Atlantic Current and transports comparatively warm waters up along the western coast of Svalbard [*Walczowski and Piechura*, 2011], while cold Arctic Ocean currents influence the eastern parts of the archipelago [*Loeng*, 1991]. Synoptic‐scale variability is mainly controlled by the interplay of extratropical cyclone activity in the south and the Arctic high pressure system in the north [*Skeie and Grønås*, 2000] and the resulting atmospheric circulation variability significantly influences the surface climate across the archipelago [*Käsmacher and Schneider*, 2011], especially during winter [*Bednorz*, 2011; *Bednorz and Fortuniak*, 2011]. During winter, northeasterly airflow dominates, while during summer, winds turn more to the southerly direction [*Käsmacher and Schneider*, 2011]. This implies that easterly weather systems approaching from the Barents Sea form the major moisture source for winter precipitation [*Førland et al.*, 1997]. Hence, sea ice conditions to the east of Svalbard exert a major influence on winter snowfall variability, since sea ice cover controls the humidity of these air masses [*Rogers et al.*, 2001; *Raper et al.*, 2005; *Zhao et al.*, 2014].

## 3 Data

For this study four different types of data are used: RCM output as climate data, repeated stake readings as in situ mass balance data, surface elevation data, and a recent glacier mask.

RCM output for the period of 2000–2011 is provided by climate fields from the European Arctic Reanalysis (EAR) product. This product was generated by *Finkelnburg* [2013] by applying the Polar Weather Research and Forecast (WRF) model (version 3.1.1) [*Hines and Bromwich*, 2008] to the Svalbard archipelago (Figure 1). Its 10 km resolution output fields (49 × 52 grid points) are created by a two‐way nesting from a higher‐level 30 km resolution domain that covers all of the European Arctic region (99 × 99 grid points). Data of this domain, in turn, are based on NCEP (U.S. National Centers for Environmental Prediction) Final Operational Global Analyses climate fields, NCEP real‐time global sea surface temperature analysis, and on AMSR‐E (Advanced Microwave Scanning Radiometer for Earth Observing System) sea ice concentration data [*Finkelnburg*, 2013]. Further information on WRF parameterizations can be found in *Finkelnburg* [2013].

**Figure 2:**
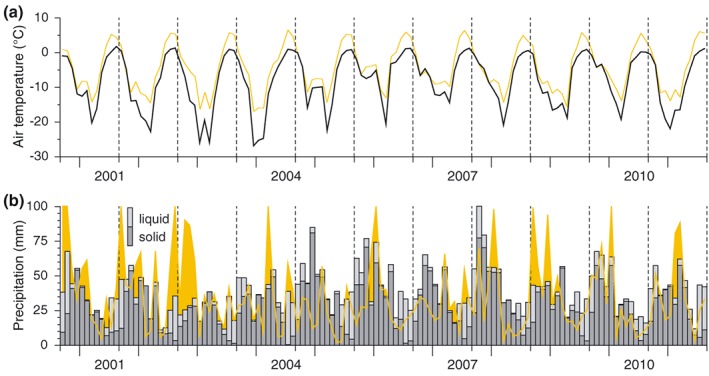
Monthly mean RCM (a) air temperature and (b) precipitation over Svalbard for the mass balance years of 2000/2001–2010/2011 (grey bars). Values represent spatial means over all grid points of the RCM domain (cf. Figure 1). Measured monthly values at the meteorological station in Ny‐Ålesund are shown for comparison (orange colors). The dashed vertical lines divide different mass balance years starting in September.

In this study we use 2 m air temperature and precipitation (total and solid) fields with a daily temporal resolution covering the period from September 2000 to August 2011 and thus the mass balance years 2000/2001–2010/2011. Figure 2 gives a basic overview of the temporal variability of the climate data over the modeling period.

A total of 517 individual stake readings, i.e., point mass balance data, were used as in situ reference. These data are unevenly distributed over four reference sites, i.e., glaciers, across the archipelago (Table 1 and Figure 1). Data from Kongsvegen and Hansbreen are based on regular seasonal measurements of summer balance in September and winter balance in April. For Nordenskiöldbreen, only annual balances based on March to April measurements are available, while for Vestfonna, there is a mix of both seasonal and annual balances. Table 1 gives an overview of stake numbers and measurement periods.

Surface topography is directly taken from the 10 km resolution surface topography that underlies the RCM calculations. Glacier areas are taken from the 2001–2010 data set in the *Glacier Area Outlines‐Svalbard* inventory [*Nuth et al.*, 2013; *König et al.*, 2014].

## 4 Methods

Our study comprises three methodical parts: (a) the setup of a CMB model for calculation of archipelago‐wide glacier CMB, (b) The adjustment of the RCM output used as forcing for the mass balance calculations, by cross validating modeled and measured mass balances, and (c) the creation of final mass balance estimates. In addition, we test the sensitivity of the results regarding different adjustment methods and critical model parameters.

### 4.1 Mass Balance Modeling

The CMB model is driven by mean daily RCM output in the form of archipelago‐wide fields of air temperature (*T*), liquid precipitation (*P_l_*), and solid precipitation (*P_s_*). The spatial resolution of the model is prescribed by the 10 km RCM output. The calculation of archipelago‐wide CMB is done by integrating overall grid cells within the model domain. Thereby, each individual grid cell is weighted according to the degree of glacier coverage (Figure 1). Our modeling is performed on a daily resolution and covers the mass balance years 2000/2001–2010/2011. The results are presented as seasonal and annual values.

Surface accumulation is taken to be equal to the amounts of *P_s_*. Surface ablation on ice and snow surfaces is calculated by applying an empirical temperature index model, i.e., degree‐day model [e.g.,*Braithwaite*, 1985] that multiplies the sum of positive mean air temperature days (we assume that no ablation occurs for days with negative mean air temperatures) with a degree‐day factor that is set to 9.0 mm w.e. K^‐1^ d^‐1^ for ice surfaces and to 7.2 mm w.e. K^‐1^ d^‐1^ for snow surfaces [*Radić and Hock*, 2011]. Differentiation between the two surface types is made possible by tracking the transient snow depth during model runs. We use degree‐day factors that are fixed in both space and time as we want our study to be entirely focused on investigating different approaches of calibrating the necessary RCM output adjustments. The sensitivity of the model to potential inaccuracies of the degree‐day factors is analyzed in the uncertainty assessment.

Refreezing is incorporated by applying the simple yet effective and well‐established *P*_max_ approach [*Reeh*, 1991; *Reijmer et al.*, 2012], in which surface melt and rain are assumed to refreeze in the winter snowpack until a predefined proportion of the total accumulation is reached. Here we use a proportion of 70% (*P*_max_ = 0.7), which is higher than the original 60% proposed by *Reeh* [1991]. Even lower values have been applied for Midtre Lovénbreen in western Svalbard [*Wright et al.*, 2007]. We increase the original value by 10% because recent studies find higher refreezing proportions on the larger ice bodies of Svalbard that are more relevant for archipelago‐wide CMB considerations. Proportions of up to 100% have been reported for Austfonna [*Østby et al.*, 2013] and Kongsvegen [*Obleitner and Lehning*, 2004], the latter representing spatially and temporally very specific conditions though. Values of 90% were successfully applied for Vestfonna in several CMB modeling studies [*Möller et al.*, 2013; *Möller and Schneider*, 2015].

### 4.2 RCM Output Adjustment

RCM air temperature and liquid and solid precipitation fields are adjusted by applying temporally and spatially fixed shifts and scalings to the entire domain. We do not consider regionally differentiated adjustments, as for example *Machguth et al.* [2013] did for Greenland's glaciers and ice caps. Our target region is much smaller, and the glacierized terrain is more heterogeneous. Furthermore, in situ measurements that could be used for calibration are only available in a limited number of representative subregions. Hence, we prefer applying an overall adjustment and accept the potential cost of corresponding uncertainties.

**Figure 3:**
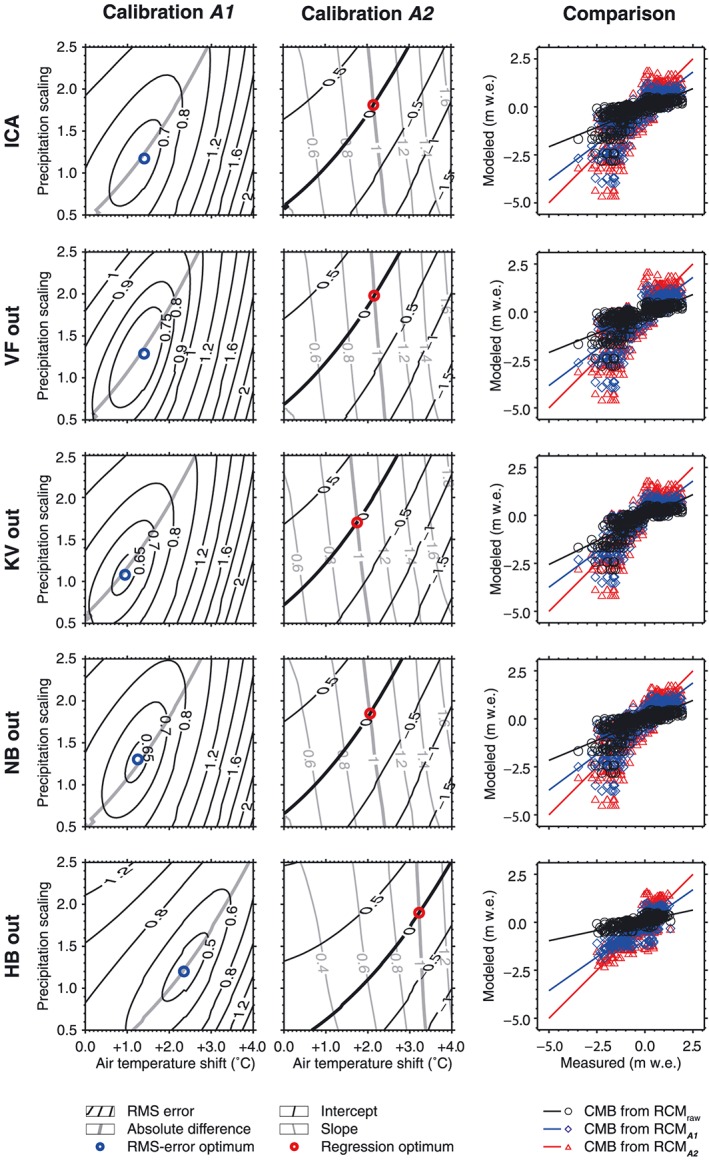
Results of (first column) calibration *_A1_* and (second column) calibration *_A2_* of the RCM output adjustments. The optimal values for each calibration procedure are marked by blue (*_A1_*) and red (*_A2_*) circles. (third column) Scatterplots represent comparisons between measured and modeled point climatic mass balances (CMB) for the raw RCM output and for adjusted RCM output (RCM_*A*1/*A*2_) as calculated for the mass balance stakes of the respective validation data set (cf. Table 2a). Results for the initial climate adjustments (ICA_*A*1/*A*2_) are shown along with the results of the four individual cross validation‐based calibration procedures (*VF out*, *KV out*, *NB out*, and *HB out*). All numbers related to this figure are given in Table 2a.

For calibration of the optimal RCM output adjustments, we run the CMB model repeatedly over a predefined range of air temperature shifts (Δ*T*) and precipitation scalings (Φ*P*). The original RCM air temperature fields are progressively altered by shifts of up to +4 K, while the RCM precipitation fields (both liquid and solid) are changed by the application of scaling factors varying between 0.5 (‐50%) and 2.5(+150%) (Figure 3). The initial optimization sequence is conducted by comparing modeled CMB to a total 517 in situ measurements at 67 mass balance stakes.

**Table 2a:** Overview of the Results of the Cross Validation and RMSE Minimization‐Based Calibration *_A1_* of the RCM Output Adjustments^a^

**ID**	***n*_cal_**	**Δ*T*_*A*1_ (°C)**	**Φ*P*_*A*1_ (None)**	***n*_val_**	**RMSE (m w.e.)**	**Intercept (m w.e.)**	**Slope (None)**	***R*^2^**
ICA_*A*1_	517	1.40	1.18	not applicable (na)	0.46	−0.06	0.75	0.64
VF out	428	1.40	1.28	89	0.15	0.33	0.67	0.66
KV out	357	0.95	1.09	160	0.49	0.20	0.36	0.72
NB out	480	1.25	1.31	37	0.73	0.63	0.61	0.63
HB out	286	2.35	1.21	231	0.97	−0.94	1.22	0.84
OCA_*A*1_	na	1.43 ± 0.47	1.23 ± 0.09	na	0.59 ± 0.35	0.16 ± 0.55	0.68 ± 0.28	0.70 ± 0.08

a The initial climate adjustment (ICA_*A*1_) is not part of the cross validation and the optimization procedure involves all *n* = 517 stake readings available. For the cross validation run *VF out*, the air temperature shift (Δ*T*_*A*1_) and the precipitation scaling (Φ*P*_*A*1_) are calibrated based on the *n*_cal_ = 428 stake readings of the calibration data set which involves data from all calibration sites except Vestfonna. The given statistical measures refer to the *n*_val_ = 89 stake readings of the validation data set involving only data from Vestfonna. For the runs *KV out*, *NB out*, and *HB* out, the allocation of stake readings to either calibration or validation data set is done analogously. For the optimal climate adjustment (OCA_*A*1_), which forms the final result of cross validation‐based calibration of the RCM output adjustment, all values given are calculated as weighted (according to *n*_cal_) mean ± one standard deviation of the four cross validation runs *VF out*, *KV out*, *NB out*, and *HB out*. The standard deviations serve as a measure of adjustment uncertainty. Units are given in parenthesis where applicable.

We use two different ways to determine the RCM output adjustment on the basis of mass balance stake data. We first minimize the root mean square error (RMSE) between modeled and measured mass balance values. The optimal result is called “*adjustment optimum A1*” (Δ*T_A1_*, Φ*P_A1_*) and the related calibration procedure is termed “*RMSE minimization (calibration _A1_)*” in the following. As an alternative, we second calculate linear regressions between the measured and the modeled values, with the optimal result being a linear fit with a slope of 1 and an intercept of 0. Here the outcome is called “*adjustment optimum A2*” (Δ*T_A2_*, Φ*P_A2_*) and the related calibration procedure is termed “*regression optimization (calibration _A2_)*.”

**Figure 4:**
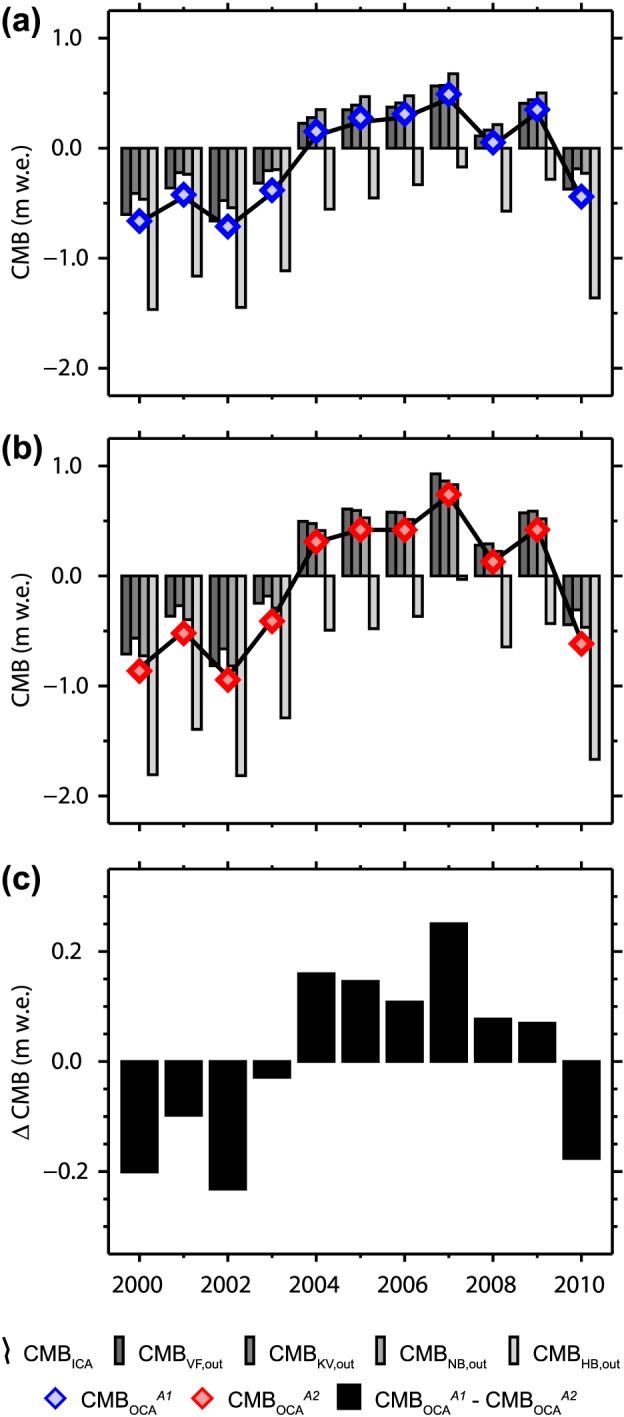
Climatic mass balance (CMB) time series for the mass balance years of 2000/2001–2010/2011, representing the outcome of (a) the RMSE minimization‐based calibration *_A1_* and (b) the regression optimization‐based calibration *_A2_* of the RCM output adjustments. Results representing the initial climate adjustment (ICA_*A*1/*A*2_) are shown as line graphs, while results of the four individual cross validation‐based calibration procedures (*VF out*, *KV out*, *NB out*, and *HB out*) are shown as bar charts. CMB time series representing the optimal climate adjustments (OCA_*A*1/*A*2_) are shown as blue (*_A1_*) and red (*_A2_*) symbols. The difference between the CMB_OCA_^*A*1^ and CMB_OCA_^*A*2^ time series is shown as additional bar chart in Figure 4c.

In a first step, we calibrate the RCM output adjustments *_A1_* and *_A2_* by using all 517 point CMB measurements available as in situ reference. The outcome of this we call the “*initial climate adjustment*” (ICA_*A*1*/A*2_). It can be interpreted as a primary adjustment, considering the maximum number of reference data in the optimization procedure. However, with no further in situ data, an independent validation of the calibration of the RCM output adjustments is not possible, and extrapolation of the results to areas beyond the calibration sites is questionable. We therefore apply a calibration method that is based on *k*‐fold cross‐validation techniques [*Kohavi*, 1995; *Möller*, 2012], in which the overall set of mass balance measurements is divided into *k* = 4 regional subsets, corresponding to the four glaciers used for calibration. The optimization procedure is then repeated *k* times. Each time one of the regional subsets is left out and Δ*T*_*A*1*/A*2_ as well as Φ*P*_*A*1*/A*2_ are determined on the basis of stake data from the remaining three calibration sites while the omitted site is used for validation and to investigate the spatial representativeness of the results. These four individual calibrations are then averaged to final calibrations for the RCM output adjustments *_A1_* and *_A2_* by weighting them according to the respective number of stake readings employed (*n_cal_*, Table 2a). We call these final adjustments the “*optimal climate adjustment*” (OCA_*A*1/*A*2_) and the corresponding RCM climate fields are henceforth termed RCM_OCA_^*A*1/*A*2^. Figure 4 presents an overview of the associated, final annual CMB time series (CMB_OCA_^*A*1/*A*2^) and all other CMB time series resulting from the different cross‐validation runs. Table 2a gives an overview of the outcome of the cross‐validated calibration in terms of air temperature shifts and precipitation scalings as well as the most important statistical measures.

### 4.3 Creation of Final CMB

**Figure 5:**
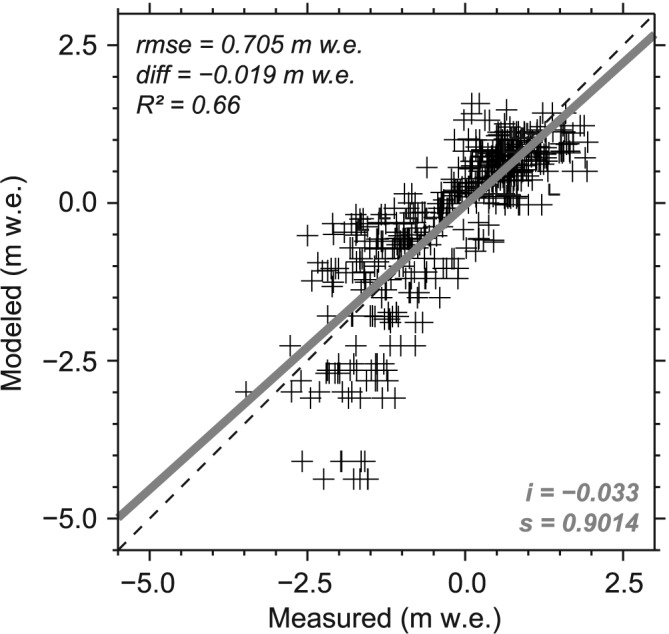
Measured point balances of all 517 individual stake readings versus their respective modeled counterparts representing the final CMB*. (top left) The statistical measures and (bottom right) the intercept (*i*) and slope (s) of the regression line (grey). The dashed line represents the one‐to‐one relation.

As a last step, the final archipelago‐wide CMB fields (CMB*) are created by forming the mean between the CMB_OCA_^*A*1^ and the CMB_OCA_^*A*2^ fields. Figure 5 shows the modeling performance related to CMB* on the basis of the 517 individual point CMB measurements. The RMS error of ±0.70 m w.e. and the negligible mean difference of ‐0.02 m w.e. suggest reliable model results. Systematic deviations remain within ±10% as indicated by an ~0.9 slope of the regression line.

The differences between CMB* and CMB_OCA_^*A*1/*A*2^ are treated as a measure for method uncertainty related to the two different ways of climate adjustment calibration.

## 5 Uncertainty Assessment

**Figure 6:**
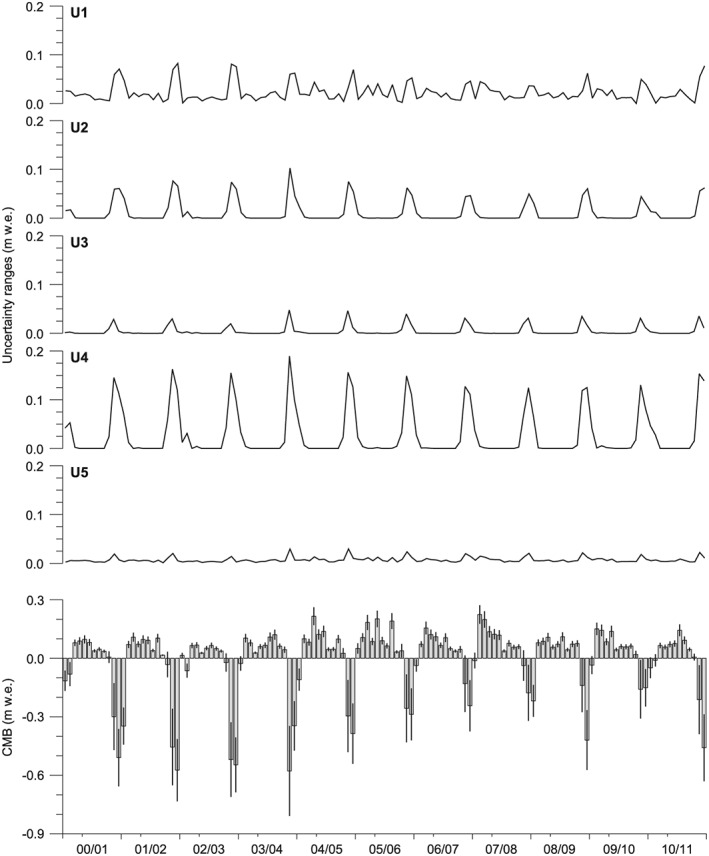
Uncertainties U* of the final monthly climatic mass balances, i.e., of (bottom) CMB*. For naming of the individual uncertainties U1–U5, refer to the text in section 5. The *x* axis shows subdivisions according to mass balance years. Starts of calendar years are indicated by minor ticks.

We quantify the overall uncertainty (U*) of the final modeled archipelago‐wide CMB (CMB*) by accounting for five different, single sources of uncertainty, resulting from RCM output adjustment and from CMB modeling. These five sources of uncertainty are assumed to be uncorrelated, so that U* is calculated by applying quadratic error propagation [*Bevington*, 1969]. Figure 6 shows an overview of monthly aggregated uncertainties.

The method uncertainty (U1) is assessed by comparing CMB* to either CMB_OCA_^*A*1^ or CMB_OCA_^*A*2^. It basically describes the spread in CMB originating from the usage of two different methods, i.e., RMSE minimization and regression optimization, for calibrating the optimal adjustments of the RCM climate fields. It is readily accessible from the original modeling procedure, i.e., without the necessity of additional model runs.

The ablation uncertainty (U2) is assessed by performing additional model runs with degree‐day factors altered according to a ±10% range, i.e., 9.0 ± 0.9 mm w.e. K^‐1^ d^‐1^ for ice surfaces and 7.2 ± 0.7 mm w.e. K^‐1^ d^‐1^ for snow surfaces. It is meant to describe the uncertainty resulting from the choice of fixed degree‐day factors.

The refreezing uncertainty (U3), i.e., the sensitivity of the simulations to the used parameterization of refreezing processes, is assessed by additional model runs, varying the *P*_max_ value by ±10%, resulting in model runs using *P*_max_ = 0.63 and *P*_max_ = 0.77, respectively. U3 is meant to describe the potential uncertainty introduced by choosing a certain, fixed *P*_max_ value for quantifying the amounts of meltwater refreezing on the basis of the sum of winter accumulation.

The uncertainties related to the applied adjustments of the driving climate variables air temperature (U4) and precipitation (U5) were finally addressed by additional model runs with air temperature shifts (Δ*T*_*A*1/*A*2_) and precipitation scalings (Φ*P*_*A*1/*A*2_) altered according to the uncertainty ranges obtained during their calibration (Table 2a). U4 and U5 thus describe the uncertainties that result from the fact that the optimal RCM output adjustments were determined using cross validation‐based techniques.

The overall uncertainty shows a clear annual cycle with uncertainties during the summer season being distinctly higher than during the winter season (Figure 6). Mean monthly U* during summer (June to August) is 0.112 ± 0.067 m w.e., while during winter (September to May), it only amounts to 0.023 ± 0.015 m w.e. The given standard deviations document that also the intermonthly variability is higher in summer than in winter. Taken together, the uncertainty assessment indicates that modeling uncertainty is between four to five times higher in the ablation season than in the accumulation season. This is because ablation uncertainties, refreezing uncertainties, and air temperature adjustment uncertainties almost exclusively affect periods with positive or close to 0°C negative air temperatures. Even the precipitation adjustment uncertainty peaks in summer due to the influence of precipitation on refreezing amounts. In this period the direct influence of rainfall and the indirect influence of increased winter accumulation overlap. Only the method uncertainty shows a diverse pattern without a clear, characteristic annual cycle.

Overall it could be stated that the uncertainties associated with the model parameters are of comparatively little importance as their absolute values are still distinctly smaller than the absolute values of modeled CMB itself (Figure 6).

## 6 Results

### 6.1 RCM Output Adjustments

The RMSE minimization‐based calibration of ICA_*A*1_ results in an air temperature shift of +1.40°C and a precipitation scaling factor of 1.18 (Figure 3). The associated modeled mass balances reach an RMSE of 0.46 m w.e. with an *R* ^2^ of 0.64 (Table 2a). The slope of 0.75 between measured and modeled balances indicates an underestimation of the absolute values of negative and positive CMB; i.e., the observed mass balance gradient is steeper than that modeled. From the cross validation‐based calibration of OCA_*A*1_, shift and scaling are similar at +1.43 ± 0.47°C and 1.23 ± 0.09 (Table 2a). Hence, shift and scaling of ICA_*A*1_ lie clearly inside the one sigma uncertainty range of the shift and the scaling calibrated for OCA_*A*1_. This indicates full comparability of both adjustments. As expected, OCA_*A*1_ leads to less accurately modeled CMB. The RMSE increases to 0.59 ± 0.35 m w.e. and the slope between measured and modeled mass balances becomes even smaller (0.68 ± 0.28) than for model forcing by RCM output corrected according to ICA_*A*1_. The explained variance, however, increases to 70 ± 8% (*R* ^2^ = 0.70 ± 0.08).

The regression optimizations (calibrations *_A2_*) result in distinctly larger RCM output adjustments. Air temperature needs to be shifted by +2.15°C (+2.22 ± 0.49°C) and precipitation needs to be scaled by 1.81 (1.86 ± 0.10) to yield optimum linear regression for ICA_*A*2_ (OCA_*A*2_) (Table 2b and Figure 3).This gives distinctly steeper mass balance gradients, with slopes between measured and modeled mass balances by design close to the optimal slope of 1.00 (0.97 ± 0.34) under ICA_*A*2_ (OCA_*A*2_) forcing. With this calibration it can be assured that positive (negative) mass balances are not affected by any systematic climate forcing‐induced underestimations (overestimations). However, this is achieved at the expense of higher RMSE compared to calibration *A*1. The percentage of variance explanation by CMB_OCA_^*A*2^ values is similar to that by CMB_OCA_^*A*1^ values. As for calibration *A*1, the ICA_*A*2_ shift and scaling lie well within the one sigma uncertainty ranges of the OCA_*A*2_ shift and scaling (Table 2b), indicating full comparability between ICA_*A*2_ and OCA_*A*2_.

**Table 2b:** Overview of the Results of the Cross Validation and Regression Optimization‐Based Calibration *_A2_* of the RCM Output Adjustments^a^

**ID**	***n*_cal_**	**Δ*T*_*A*2_ (°C)**	**Φ*P*_*A*2_ (None)**	***n*_val_**	**RMSE (m w.e.)**	**Intercept (m w.e.)**	**Slope (None)**	***R*^2^**
ICA_*A*2_	517	2.15	1.81	na	0.57	0.00	1.00	0.66
VF out	428	2.15	1.98	89	0.35	0.52	1.01	0.67
KV out	357	1.75	1.71	160	0.43	0.31	0.55	0.72
NB out	480	2.05	1.84	37	0.82	0.86	0.88	0.64
HB out	286	3.20	1.88	231	1.37	−1.14	1.58	0.86
OCA_*A*2_	na	2.22 ± 0.49	1.86 ± 0.10	na	0.74 ± 0.47	0.27 ± 0.70	0.97 ± 0.34	0.71 ± 0.08

a The layout of the table fully accords to the one of Table 2a.

When comparing the performances of modeled mass balances resulting from climate forcing according to RCM_ICA_^*A*1/*A*2^ and RCM_OCA_^*A*1/*A*2^, it turns out that they all lie close to each other. As expected, given the targets of the two calibration procedures, RMSE values are smaller in the RMSE minimization scheme (calibration *A*1) while the slope is close to perfect under the influence of the regression optimization scheme (calibration *A*2). Apart from the different targets during calibration, unforeseen impacts of the CMB model architecture might also have contributed to these differences in quality measures.

### 6.2 Mass Balances

The calculated annual cycle of the archipelago‐wide CMB of the glaciers and ice caps of Svalbard suggests a long accumulation period that typically lasts from September to May and a rather short ablation period that is mostly limited to the summer months June, July, and August (Figure 6). Despite this considerable difference in duration, the absolute amounts of winter balances (+0.58 ± 0.15 m w.e. a^‐1^) and summer balances (‐0.63 ± 0.33 m w.e. a^‐1^) are comparable, leading to an only slightly negative mean rate of ‐0.05 ± 0.40 m w.e. a^‐1^ for the archipelago‐wide annual CMB over the mass balance years 2000/2001–2010/2011 (Table 3). The associated mean equilibrium line altitude (ELA) is 452 ± 200 m asl.

**Table 3:** Key Figures and Mean Final Climatic Mass Balance (CMB*) Values for Nine Different Subregions of the Svalbard Archipelago^a^

**Subregion**	**Location**	**Area(km^2^)**	**Elevation(m asl)**	***B_w_* (m w.e.)**	***B_s_* (m w.e.)**	***B_a_* (m w.e.)**	**ELA (m asl)**
1*	NW Spitsbergen	5,258	410	+0.53 ± 0.13	−0.69 ± 0.34	−0.16 ± 0.38	505 ± 126
2	Andrée & Dickson Land	965	537	+0.48 ± 0.12	−0.73 ± 0.32	−0.25 ± 0.36	634 ± 193
3*	NE Spitsbergen	8,501	547	+0.71 ± 0.16	−0.50 ± 0.28	+0.22 ± 0.36	535 ± 241
4	Nordenskiöldland	655	363	+0.46 ± 0.15	−1.24 ± 0.42	−0.78 ± 0.50	547 ± 86
5*	S Spitsbergen	4,753	327	+0.62 ± 0.20	−0.90 ± 0.41	−0.28 ± 0.53	394 ± 81
6*	Vestfonna	2,389	345	+0.49 ± 0.12	−0.47 ± 0.29	+0.03 ± 0.33	337 ± 107
7	Austfonna	8,315	327	+0.53 ± 0.13	−0.54 ± 0.31	0.00 ± 0.37	325 ± 141
8	Barentsøya and Edgeøya	2,288	227	+0.57 ± 0.17	−0.76 ± 0.40	−0.19 ± 0.49	374 ± 153
9	Kvitøya	646	6	+0.30 ± 0.09	−0.45 ± 0.39	−0.15 ± 0.45	na
All	na	33,770	390	+0.58 ± 0.15	−0.63 ± 0.33	−0.05 ± 0.40	452 ± 200

a A map of the spatial distribution of the nine subregions is given in Figure 7f. The glacierized area within each subregion is shown along with its mean elevation in the RCM topography. Glacierized area‐wide winter (*B_w_*), summer (*B_s_*), and annual (*B_a_*) CMB and the associated equilibrium line altitudes (ELA) are given as averages over the mass balance years of 2000/2001–2010/2011 together with their respective U* uncertainty ranges. Spatial averaging of CMB is done by weighting the values of individual grid points according to the associated glacier area (cf. Figure 1). For subregion nine, no ELA could be derived because of limitations in the RCM elevation information in this area. Subregions with calibration sites (cf. Figure 1) are marked with an asterisk.

Clear temporal variability is evident over the modeling period. While the first half of the decade is dominated by negative annual balances, predominantly positive annual balances prevail over the second half (Figure 7). Year 2001/2002 appears to be the most negative (‐0.81 ± 0.40 m w.e.) mass balance year in the modeling period and 2007/2008 the most positive (+0.80 ± 0.37 m w.e.). Accordingly, the ELA varies from 957 ± 190 m asl for the 2001/2002 mass balance year to 187 ± 120 m asl for 2007/2008 (Figure 7). These extremes not only depend on extremes of either winter or summer balance but on a distinct combination of the two. The mass balance year 2001/2002 shows the least positive winter balance in combination with the second most negative summer balance, while in 2007/2008 the situation is exactly vice versa.

**Figure 7:**
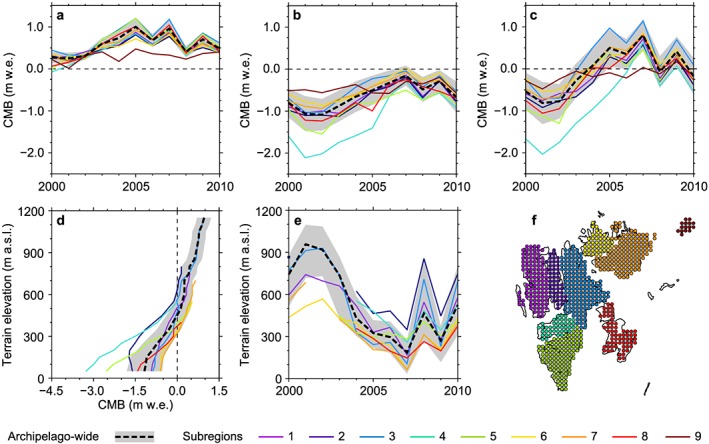
Modeled glacier‐wide final climatic mass balances, i.e., CMB*, for nine different subregions of the Svalbard archipelago (color‐coded lines). The archipelago‐wide means for the entire Svalbard (black, dashed line) are given together with their uncertainty ranges (grey shading). Results are displayed for the mass balance years of 2000/2001–2010/2011. (a) Winter balances, (b) summer balances, and (c) annual balances are shown together with (d) the mean mass balance profiles and (e) the equilibrium line altitude (ELA) variability. (f) A map of the nine subregions as represented by the grid points of the RCM.. Subregion numbers correspond to those in Table 3. Spatial averaging of climatic mass balance is done by weighting the values of individual grid points according to the associated glacier area (cf. Figure 1).

Given the regional climate developments over the modeling period (Figure 2), it is obvious that the transition from prevailingly negative mass balance years during the first half of the decade to rather balanced or even positive years during the second half is associated with an increase of air temperature in winter and a simultaneous decrease in summer. The average minimum air temperatures of winters during 2006–2011 (−15.6 ± 3.9°C) were distinctly less negative than those of the earlier 2001–2005 period (−20.8 ± 4.5°C). This change in winter air temperature was accompanied by an increase in winter precipitation of ~40% which, in turn, could be seen as the direct driver for the more positive winter balances during the second half of the modeling period. Summer air temperatures, in contrast, show a significantly negative trend over the modeling period. Mean monthly values were close to 0.9°C in the beginning of the decade and dropped to below 0.2°C at its end, which decreased ablation amounts.

**Figure 8:**
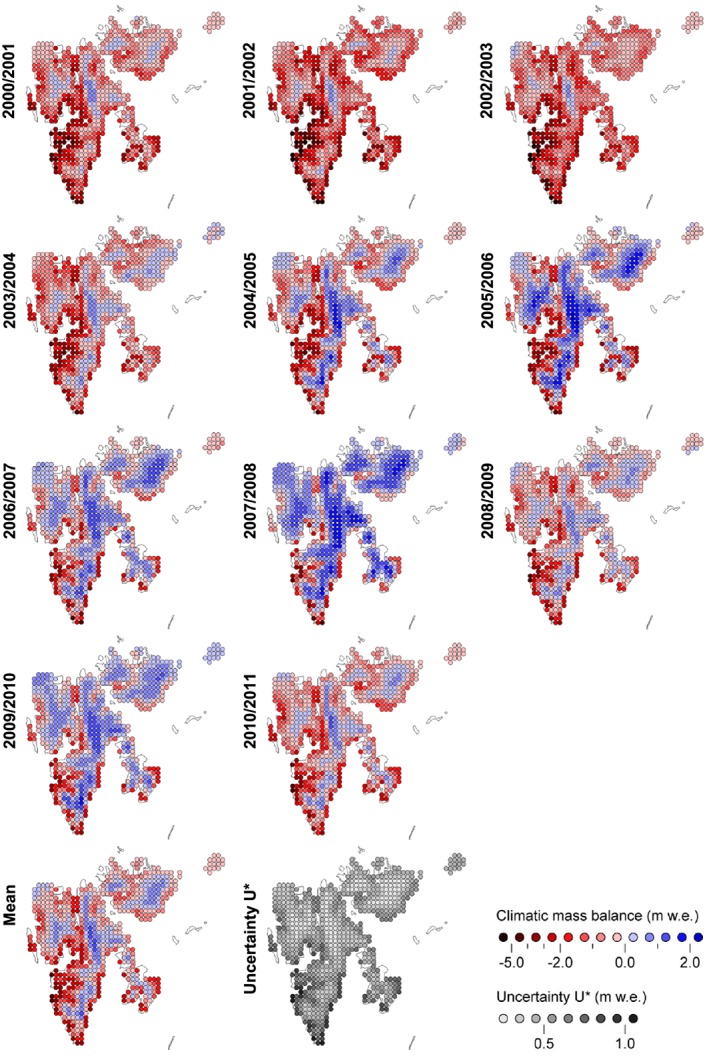
Annual climatic mass balance fields for the Svalbard archipelago over the period of 2000/2001–2010/2011, representing the final modeling results (CMB*). (bottom right) The mean CMB* field over the modeling period including the associated uncertainty range field (U*, cf. Figure 6).

Also the spatiotemporal variability of CMB across the archipelago and over the nine subregions is considerable (Figures 7 and 8). In the most negative mass balance year (2001/2002), accumulation areas are limited to the very highest parts of NW, NE, and S Spitsbergen and to the central parts of Vestfonna and Austfonna. During the most positive mass balance year (2007/2008), in contrast, all subregions show extensive areas with net accumulation (Figure 8) and ablation is mostly limited to the nearshore areas of the southern half of Svalbard. When looking at the mean CMB over the modeling period, all subregions feature areas of positive mass balance and thus a mean ELA well within the range of glacierized elevations (Figure 8 and Table 3). However, subregions 2 and 4 are still predominantly characterized by negative CMB areas. The most positive specific balances are consistently observable in the uppermost parts of central NE Spitsbergen, while the most negative specific balances are always found along the southwestern coasts of the archipelago (Figure 8).

Taking all these observations together, a characteristic spatial variability of CMB across Svalbard becomes obvious that is reconfirmed in the spatial variability of the ELA (Table 3). While the ELAs across the northern and central parts of Spitsbergen (subregions 1–4) lie clearly above the archipelago‐wide mean, the ELAs on the eastern islands and in southern Spitsbergen (subregions 5–8) lie below. Hence, the ELA shows an increase from southeast to northwest. This general pattern is, in addition, superimposed by a clearly observable tendency toward even higher ELAs in the interior parts of Spitsbergen, i.e., in subregions 2 and 4.

This characteristic pattern can be explained by an interplay of two different effects. In general, the states of subregion‐wide CMB across Svalbard either show dependencies on climate or on hypsometry. The climate of a subregion governs the amounts of ablation and accumulation and thus the shape of the CMB profile and the altitude of the equilibrium line. The hypsometry of a subregion, however, controls its accumulation area ratio and thus the relation of areas above and below the equilibrium line. This means, that subregions with a similar climate can show substantially different CMB due to different hypsometric characteristics.

NE Spitsbergen emerges as the subregion with clearly the most positive CMB (Figure 7 and Table 3) at an annual mean of +0.22 ± 0.36 m w.e. a^−1^ over the modeling period. Even though the mean ELA of this subregion (535 ± 241 m asl) lies clearly above the archipelago‐wide average, it is situated far below the highest elevations within this area, resulting in an extensive mean accumulation area that covers considerable parts of this second largest contiguous ice mass of Svalbard (Figure 8). This indicates that the positive balances in subregion 3 are governed by favorable glacier hypsometry rather than by climate.

In contrast, a distinctly stronger climate dependency is observable for the two large ice caps of Svalbard, Vestfonna (subregion 6; +0.03 ± 0.33 m w.e. a^−1^) and Austfonna (subregion 7; +0.00 ± 0.37 m w.e. a^−1^), which are characterized by more or less balanced CMB. Despite their rather limited extent into high elevations, the lowest ELAs of the archipelago (Table 3 and Figure 7e) lead to the fact that their extensive central plateaus are located inside the accumulation zone, which prevents them from being rather ablation controlled. Taken together, the balanced states of the two ice caps have to be attributed to both local climate favor and a hypsometry, which only becomes favorable due to the positive climate influence.

Nordenskiöldland clearly shows the most negative CMB (−0.78 ± 0.50 m w.e. a^−1^) of all subregions (Table 3). Except for the most positive year (2007/2008), its annual balances are distinctly more negative than those of all other subregions (Figure 7). The mean ELA of this subregion 4 (547 ± 86 m asl) is the second highest of all over Svalbard (Table 3) but is still comparable to that of the strongly positive subregion 2. However, unlike in NE Spitsbergen, in Nordenskiöldland, almost no glacierized areas extend into regions above the ELA. This suggests, that the strongly negative balances of subregion 4 mainly result from an unfavorable combination of glacier hypsometries and regional climate conditions.

In Andrée Land and Dickson Land (subregion 2), the effect of regional climate disadvantage for glacier mass balance becomes even more pronounced. Despite the fact that the glacierized areas within this subregion extend into elevations which are the second highest of all nine subregions, the mean annual CMB profile shows almost exclusively negative balances (Figure 7). This conforms to the fact that mean ELA of this subregion (634 ± 193 m asl) is by far the highest on the archipelago (Table 3). Hence, the persistently negative CMB (−0.25 ± 0.36 m w.e. a^−1^) of Andrée Land and Dickson Land can be interpreted as being predominantly climate driven.

## 7 Discussion

### 7.1 RCM Output Adjustment

**Figure 9:**
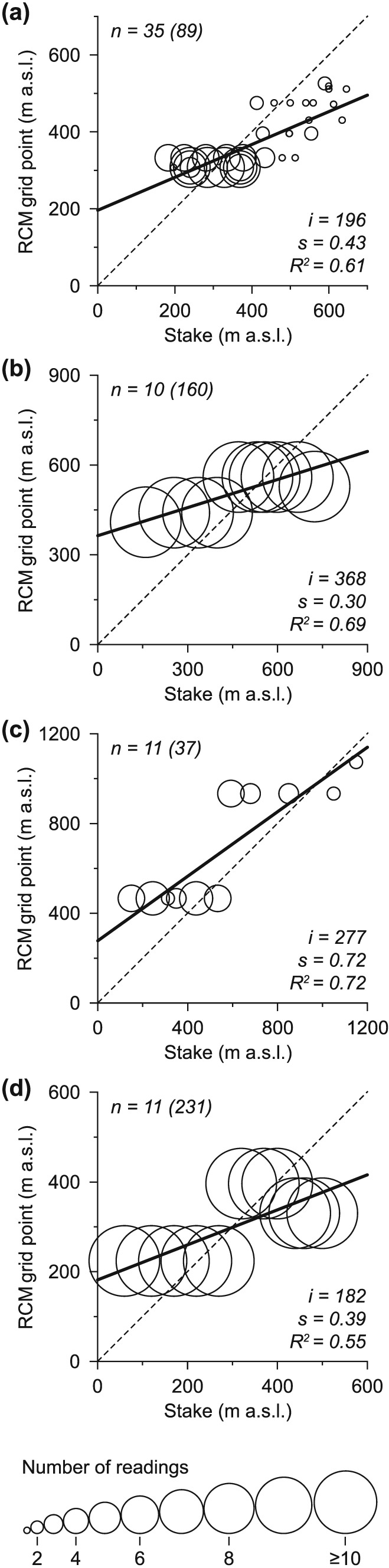
Comparison between elevations of individual stake locations and of associated RCM grid points at (a) Vestfonna, (b) Kongsvegen, (c) Nordenskiöldbreen, and (d) Hansbreen, with the number of measurements at each stake indicated by bubble size. Note that at Kongsvegen each stake has been measured 16 times and at Hansbreen 21 times. (top left) The number of stakes (*n*) and the total number of all measurements (in parenthesis). The black line represents a linear fit weighted according to the number of measurements at each stake. (bottom left) The intercept (*i*), slope (*s*), and *R* ^2^ of the fit.

As outlined above, the RCM output adjustments are considerably different and calibration optima *A*1 and *A*2 are thus hardly comparable. This is because of unevenly distributed stake locations and measurement frequencies (Figure 9) as well as measurement periods (Table 1). Distinct parts of the elevation range are overrepresented in the in situ‐measured mass balance data. The number of measurements per stake varies strongly across the network, and the temporal extent of the point balance time series is also inhomogeneous. This leads to considerable spatiotemporal disparities in the point balance data set which forms the in situ reference of the calibration procedure.

The RMSE minimization‐based calibration scheme (calibration *A*1) is predominantly based on spatially and temporally overrepresented elevations, which leads to mass balance estimates that agree well within the regions showing similar terrain elevations as the in situ data. However, outside of these regions, accuracy can be expected to decreases considerably with increasing elevational distance and decreasing measurement frequencies.

The fact that the calibration procedures are based on a comparison of modeled CMB values at RCM grid elevations and measured CMB values at real‐world stake elevations also induces strong influences on the regression optimization‐based calibration scheme (calibration *A*2), which mainly aims at adequately reproducing the CMB gradient, by avoiding underestimation of the absolute values of negative and positive CMB. Since CMB varies predominantly with elevation, the considerable deviations between RCM and real‐world topography lead to an erroneous tilt in the modeled CMB gradient. Given that low elevations are overestimated in low‐resolution topographies while higher ones are underestimated [e.g.,*Paul*, 2008], this in turn results in RCM output adjustments that tend to be too high in terms of absolute values of both air temperature and precipitation.

Taken together, each of the two calibration schemes is indeed negatively influenced by the limitations of combined representativeness of model topography and in situ measurements, but they nevertheless lead to optimal results regarding one of the two calibration methods. Hence, it can be expected that the averaging of CMB_OCA_^*A*1^ and CMB_OCA_^*A*2^ for generation of the final CMB* fields forms a suitable way to mediate between the two requirements of an optimal climate‐adjustment calibration, i.e., RMSE minimization and regression optimization.

The individual shifts (scalings) of the cross validation‐generated OCA_*A*1_ show large spatial differences and range between +0.95°C (1.09) when leaving out data from Kongsvegen during calibration and +2.35°C (1.31) when leaving out data from Hansbreen (Nordenskiöldbreen) (Table 2a). In the calibration of OCA_*A*2_, these ranges are stretched between +1.75°C (1.71) when leaving out data from Kongsvegen and +3.20°C (1.98) when leaving out data from Hansbreen (Vestfonna) (Table 2b). These spreads of the calibrated climate adjustments suggest that the original RCM air temperature and precipitation fields show regionally diverse inaccuracies that prohibit a better performance of CMB modeling. The only way of improving the modeling performance on the basis of pure climate adjustments could be a spatially distributed calibration of the shifts and scaling factors. However, a clear, archipelago‐wide spatial pattern cannot be derived from four calibration sites only, and interpolations or extrapolations of individual calibrations at these sites to the entire archipelago are thus not feasible. Such would require significantly more sites with in situ mass balance measurements in order to allow for a better regional diversification of the calibration procedure and for a more dense net of tie points for extrapolation. In addition, spatially varying degree‐day factors and refreezing could be considered in the CMB model. However, this again would require more and better distributed in situ measurements for model calibration.

### 7.2 Mass Balances

The overall picture of CMB variability across the archipelago over the modeling period is in accordance with results from earlier, archipelago‐wide studies [e.g.,*Hagen et al.*, 2003; *Moholdt et al.*, 2010b] and it also conforms to recent findings by *Lang et al.* [2015] who derived a modeled surface mass balance of entire Svalbard from calculations with the regional climate model MAR.

The spatial variability of the ELA derived in our study closely resembles the ELA distribution presented by *Hagen et al.* [2003]. A general increase from southeast to northwest is superimposed on a substantial increase toward the interior parts of Spitsbergen with the highest ELA being present in Andrée Land and Dickson Land, i.e., in our subregion 2. The only major difference occurs on Vestfonna where our ELA estimate is about 200 m lower than the earlier one of *Hagen et al.* [2003]. This lower estimate is backed by a detailed and well‐founded modeling study of *Möller et al.* [2013] who derived a mean ELA of 354 m asl over our 2000/2001 to 2010/2011 modeling period, which lies well within the uncertainty range of our only slightly lower ELA estimate (337 ± 107 m asl). However, it has to be noted that the ELA of individual mass balance years shows a much stronger variability in this study (between 61 and 567 m asl) than in the one of *Möller et al.* [2013] (between 281 and 454 m asl).

For the period of 2003–2008, *Moholdt et al.* [2010b] obtained a mean annual geodetic balance of −0.12 ± 0.40 m w.e. a^−1^ for entire Svalbard, while our modeling yields a CMB rate of +0.23 ± 0.40 m w.e. a^−1^ over the same period. As the geodetic balance also includes calving losses at marine‐terminating glacier margins, this value should certainly be more negative than the contemporaneous CMB. Assuming a mean annual calving contribution to the overall mass balance of −0.20 ± 0.05 m w.e. a^−1^ (−6.75 ± 1.7 km^3^ w.e. a^−1^ [*Błaszczyk et al.*, 2009]), the remote‐sensing‐based observations of *Moholdt et al.* [2010b] suggest an archipelago‐wide mass balance of +0.08 m w.e. a^−1^, excluding calving losses. However, as the calving flux estimate of *Błaszczyk et al.* [2009] excludes losses from Kvitøya and as geodetic balances do not account for mass gain through refreezing processes, it is reasonable to assume that this value has to be corrected even further into the positive direction to finally arrive at a reliable estimate for the CMB of entire Svalbard. Hence, the observation‐based, geodetic mass balance estimate of *Moholdt et al.* [2010b] even further approaches our modeled CMB, which, however, is still slightly more positive.

The modeling results of *Lang et al.* [2015] suggest predominantly negative mass balances for the first half of our study period and mostly positive balances for its second half. This is in accordance with our findings. However, *Lang et al.* [2015] obtained a range of individual annual balances roughly between −0.35 m w.e. a^−1^ and +0.20 m w.e. a^−1^, while our balances range between −0.81 m w.e. a^−1^ and +0.80 m w.e. a^−1^. Nevertheless, the mean annual balances over the period of 2000/2001–2010/2011 are rather similar at ~−0.10 m w.e. a^−1^ [*Lang et al.*, 2015] and −0.05 ± 0.40 m w.e. a^−1^ in our study. This means that, while the average balances are similar, their absolute annual rates are larger in our study. In addition, also the mass balance profiles (Figure 7d) show very good agreement between both studies across most subregions of the archipelago. Just the lowermost parts of the profiles in areas of rather discontinuous glacier coverage, i.e., especially Nordenskiöldland and the coastal parts of S Spitsbergen, tend to be considerably more negative in our study. This suggests the assumption that the manner of creation of the *Lang et al.* [2015] ice mask leads to an underestimation of absolute ablation amounts and thus to a positive bias in surface mass balance in the respective areas.

Differences in the ice masks of the two RCM make our archipeleago‐wide balances not directly comparable to those of *Lang et al.* [2015]. While our analysis includes Kvitøya, the study of *Lang et al.* [2015] does not. Furthermore, while their study is also based on a 10 km grid, it only considers cells with more than 50% glacier coverage but assumes them to be 100% covered. The more discontinuous glacierized areas, that result in 10 km grid cells with less than 50% glacier coverage, are almost exclusively situated at the outer, and thus lower, margins of the ice masses. Hence, by excluding these grid cells, parts of the ablation zones across Svalbard are not accounted for, which leads to a small positive bias in archipelago‐wide ablation sums. This, in turn, drives the mean balances derived in the two studies slightly farther away from each other. However, the average surface mass balance obtained by *Lang et al.* [2015] does still lie well within the uncertainty bounds of our average CMB estimate.

**Figure 10:**
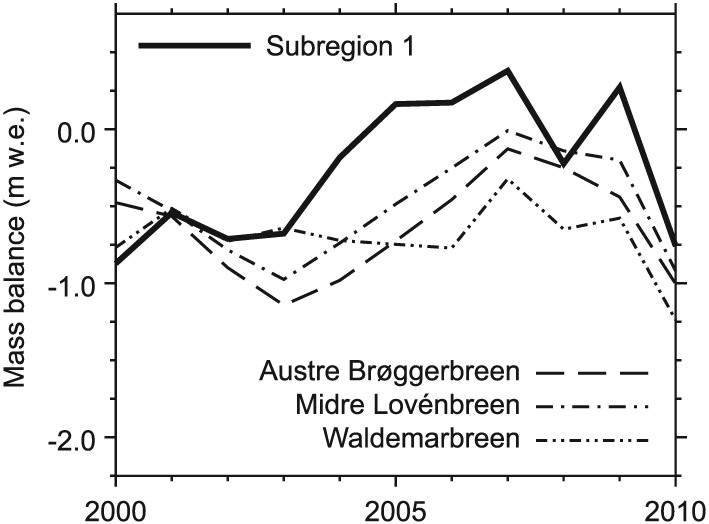
Modeled annual CMB of NW Spitsbergen (subregion 1, cf. Figure 7f) compared to measured annual balances from three individual glaciers within this subregion. Data for Waldemarbreen were provided by the World Glacier Monitoring Service.

Independent in situ observations over the modeling period that are available from the World Glacier Monitoring Service for three glaciers in subregion 1 (Austre Brøggerbreen, Midtre Lovénbreen, and Waldemarbreen) likewise support our model results. The interannual variabilities of the measured annual balances of these glaciers show a similarity to the variability within our modeled CMB for this subregion (Figure 10). Indeed, the modeled CMB is more positive, but this can be attributed to the fact that the measured glaciers are low‐lying valley glaciers that do not feature extensive accumulation areas like subregion 1 as a whole.

When comparing the modeled CMB time series with detailed modeling studies for individual glaciers or ice caps, an inconsistent picture emerges. Comparing modeled CMB in subregion 7 with measured balances of a northwesterly basin of Austfonna ice cap [*Moholdt et al.*, 2010a] shows good agreement for the trend over the period of 2004–2008, where both modeling and measurements suggest increasing annual balances and decreasing ELAs. While *Moholdt et al.* [2010a] found balances from roughly −0.5 m w.e. a^−1^ to +0.5 m w.e. a^−1^ for the northwesterly basin, our study suggests ice cap‐wide balances between −0.12 m w.e. a^−1^ and +0.81 m w.e. a^−1^ (Figure 7). The considerable positive bias of our results can, however, at least partly be attributed to differences in hypsometry, with Austfonna ice cap as a whole showing a considerably larger share of accumulation‐dominated areas than it is the case for the basin only. A comparison of the modeled CMB time series of subregion 6 with the results of more detailed and dedicated modeling studies for Vestfonna ice cap [*Möller et al.*, 2011, 2013], in contrast, reveals considerable discrepancies except for the above described positive CMB trend over the period of 2004–2008 which is clearly observable. The range of annual glacier‐wide CMB obtained in our study is distinctly larger than the one obtained by *Möller et al.* [2013]; i.e., positive CMB are more positive while negative CMB are more negative. For the period of 2000/2001–2010/2011, annual CMB in our study range between −0.55 m w.e. and +0.84 m w.e. (Figure 7) while in *Möller et al.* [2013], a spread between −0.25 m w.e. and +0.27 m w.e. is presented. Nevertheless, the annual CMB gradients for Vestfonna are about twice as steep in *Möller et al.* [2013] than in our study (Figure 7). This finding contradicts the archipelago‐wide good agreement between the mean surface mass balance profiles obtained by *Lang et al.* [2015] and the CMB profiles presented here (Figure 7). While a discrepancy exists compared to the findings of *Möller et al.* [2013], our results are in accordance with those obtained by *Lang et al.* [2015].

Several other mass balance studies at various glaciers across Svalbard [e.g.,*Karner et al.*, 2013; *Sobota*, 2011; *van Pelt et al.*, 2012] also suggest a generally positive trend of surface and climatic mass balance over the first decade of the 21st century and thus support our modeling results (Figure 7). However, toward the end of the decade, rather inconsistent pictures are drawn by the individual studies, indicating diverging regional mass balance evolution across the archipelago, which is not in line with our findings (Figures 7 and 8).

The latter suggests that spatially variable adjustments of climate data are needed to compensate for eventual regionally limited inaccuracies in RCM climate fields. The revealed discrepancies also suggest that the calibration of a CMB model with spatially homogeneous parameters does not per se facilitate a modeling that yields equally reliable results for all subregions of Svalbard even if stakes from several different locations on the archipelago are used as in situ reference. This is because of the fact that the characteristics of the local mass balance regimes of the rather small calibration sites are extrapolated to the entire archipelago. Hence, this again calls for the incorporation of spatially distributed model parameters and the consideration of as much and as equally distributed as possible in situ reference data for calibration.

Overall, it could be stated that there are regionally varying differences between our modeling results and other modeled or measured mass balances. This might be attributed to (a) our use of spatially constant adjustment parameters for the RCM output, (b) the substantial elevation biases introduced by the coarse resolution climate grids, or (c) the shortcomings introduced by using a temperature index model for calculation of ablation. It should also be borne in mind that our CMB estimates are not directly comparable to the estimates presented in several other mass balance studies, as the latter partly present surface mass balances instead of climatic mass balances and thus neglect refreezing below the previous year's end‐of‐summer surface. Moreover, additional inaccuracy might be introduced by spatially and temporally varying thermal regimes of the ice masses or other glacier‐related issues that are not explicitly accounted for in the model such as, e.g., snow redistribution by wind or calving processes. Nevertheless and despite showing a tendency toward too positive values, our modeling results can be seen as a reliable estimate for recent CMB variability across the Svalbard archipelago given an adequate consideration of the uncertainty ranges.

Finally, it has to be borne in mind that our results for Kvitøya (subregion 9) have to be considered with special caution because of limitations in the RCM elevation data. While the ice cap of Kvitøya rises to an elevation of 256 m asl, it only has maximum elevations of less than 10 m asl in the RCM topography. This fact can be expected to have a severe impact on the RCM climate data from this area. When looking at the CMB in subregion 9, it becomes obvious that Kvitøya is the only subregion that does not resemble the average interannual variability of CMB over the modeling period (Figure 7). Given the spatial proximity of all subregions, this observation rises doubts about the reliability of our modeled CMB for subregion 9. In addition, ELAs cannot be derived for this region.

## 8 Conclusion

The CMB of all glacierized areas on Svalbard was modeled for the mass balance years 2000/2001–2010/2011. The resulting mean annual CMB over the modeling period is −0.05 ± 0.40 m w.e. a^−1^ and the average ELA is 452 ± 200 m asl. The central part of Svalbard, i.e., Nordenskiöldland (subregion 4), shows the most negative CMB, while the highest parts of the archipelago in northeastern Spitsbergen (subregion 3) show the most positive. The temporal evolution of CMB is characterized by predominantly negative mass balance years over the first half of the decade and by rather positive mass balance years over its second half.

The derived uncertainty of modeled CMB is characterized by considerable intraannual variability, which is induced by a complex interplay of individual uncertainties related to various sources in model calibration and application. The overall uncertainty mainly concentrates on the ablation‐dominated summer months as individual uncertainties related to RCM air temperature adjustment and degree‐day factor choice have by far the strongest impact. Moreover, substantial regional disparities and an overall elevational variability of the final uncertainty range with larger uncertainties in low‐lying and smaller uncertainties in high‐lying areas are evident.

The model used to calculate the archipelago‐wide CMB was forced by spatially distributed RCM output, i.e., 10 km horizontal resolution air temperature and precipitation fields. The suitability of this coarse resolution RCM output for deriving reliable region‐wide CMB was evaluated.

We applied an archipelago‐wide homogeneous adjustment of RCM output in form of air temperature shifts and precipitation scalings in order to achieve the best possible CMB results for the entire Svalbard archipelago, as validated by a data set of several hundreds of measured point mass balances. The cross‐validated calibration of these RCM output adjustments, however, revealed considerable regional differences. Air temperature shifts and precipitation scalings show considerable variability across the different cross validation runs. Moreover, considerable differences regarding the necessary adjustments occurred depending on whether RMSE minimization or regression optimization was used as quality measure during the calibration procedure.

We further find that the uncertainty of the coarse resolution RCM output suggests a necessity for higher resolution spatially distributed downscaling of the climate data required for reliable model forcing. Insufficient representation of surface topography in the regional climate model and inhomogeneous elevational distribution of reference mass balance measurements were identified as particularly crucial factors during model calibration. This calls for modeling climatic input at higher spatial resolution and extending observational networks to other glaciers with larger elevation range. Moreover, our results suggest that the parameters of the temperature index‐based CMB model should be calibrated as regionally or even fully spatially variable quantities to achieve improved model performance.

Nevertheless, comparisons with independent mass balance measurements or more detailed modeling studies indicates a good quality of our modeled CMB. Validation of the results confirms that they provide a reliable, although slightly too positive, representation of the CMB of Svalbard's glaciers and ice caps as a whole.
